# How can behavioural science contribute to qualitative research on antimicrobial stewardship in primary care?

**DOI:** 10.1093/jacamr/dlac007

**Published:** 2022-02-07

**Authors:** Aleksandra J. Borek, Marta Santillo, Marta Wanat, Christopher C. Butler, Sarah Tonkin-Crine

**Affiliations:** 1 Nuffield Department of Primary Care Health Sciences, University of Oxford, Oxford, UK; 2 National Institute for Health Research (NIHR) Health Protection Research Unit (HPRU) in Healthcare Associated Infections and Antimicrobial Resistance, University of Oxford, Oxford, UK

## Abstract

Antibiotic use (and misuse) accelerates antimicrobial resistance (AMR), and addressing this complex problem necessitates behaviour change related to infection prevention and management and to antibiotic prescribing and use. As most antibiotic courses are prescribed in primary care, a key focus of antimicrobial stewardship (AMS) is on changing behaviours outside of hospital. Behavioural science draws on behaviour change theories, techniques and methods developed in health psychology, and can be used to help understand and change behaviours related to AMR/AMS. Qualitative methodologies can be used together with a behavioural science approach to explore influences on behaviour and develop and evaluate behavioural interventions. This paper provides an overview of how the behavioural science approach, together with qualitative methods, can contribute and add value to AMS projects. First, it introduces and explains the relevance of the behavioural science approach to AMR/AMS. Second, it provides an overview of behaviour change ‘tools’: behaviour change theories/models, behavioural determinants and behaviour change techniques. Third, it explains how behavioural methods can be used to: (i) define a clinical problem in behavioural terms and identify behavioural influences; (ii) develop and implement behavioural AMS interventions; and (iii) evaluate them. These are illustrated with examples of using qualitative methods in AMS studies in primary care. Finally, the paper concludes by summarizing the main contributions of taking the behavioural science approach to qualitative AMS research in primary care and discussing the key implications and future directions for research and practice.

## Introduction

Antimicrobial resistance (AMR) is a well-recognized urgent public health priority.^1–3^ The COVID-19 and earlier pandemics have shown us the challenges and dangers to individuals and societies globally when faced with a lack of effective treatments for infections. Worldwide, over 700 000 people die each year from drug-resistant infections and AMR is projected to cause 10 million deaths annually, becoming the leading cause of death by 2050, unless action is taken to avert it.^4^ While AMR is influenced by a variety of factors and requires multifaceted and multidisciplinary approaches, antibiotic over-prescribing and overuse are among the biggest modifiable contributors.^3,4^ Most antibiotics are prescribed in primary care,^5^ often inappropriately^6^ (i.e. not according to guidelines and for infections that do not usually benefit from antibiotics). Thus, optimizing antibiotic prescribing in primary care is a particularly important target for antimicrobial stewardship (AMS) programmes/interventions.

Human behaviours (actions) play an important role in both driving and mitigating AMR. As behaviours, they are influenced by factors and processes that shape all human behaviour. While multidisciplinary approaches are necessary due to the complexity of AMR and AMS, behavioural science can help with understanding and changing relevant behaviours of the public, patients, professionals and organizations. Understanding behaviour and promoting behaviour change related to AMR/AMS have been recommended in the global and national action plans to address AMR.^2–4^ The role and contribution of behavioural science and behaviour change in AMR/AMS have also been increasingly recognized in research.^7–9^ Growing evidence shows that behavioural interventions are effective in addressing AMR/AMS related behaviours (e.g. reducing clinically unnecessary antibiotic prescribing), thus supporting the use of behavioural science.^10–13^

Behavioural science stretches across multiple fields. In this paper, by a ‘behavioural science approach’ we specifically mean an approach to understanding and changing health-related behaviours that draws on the research on psychological and behaviour change developed in health psychology. It offers a wealth of health-related behaviour change ‘tools’ (e.g. behaviour change theories, techniques), methods (e.g. to develop behavioural interventions) and evidence (e.g. on behaviour change techniques and interventions). The behavioural science approach often involves using qualitative methodologies and methods because they are particularly suitable to identify influences on behaviour and help develop and evaluate interventions, and thus both can be used together in AMS research (M. Wanat, M. Santillo, A. J. Borek, C. C. Butler, S. Tonkin-Crine, ‘The value, challenges and practical considerations when conducting qualitative research on antimicrobial stewardship in primary care’, under review).^14^

In this paper we aim to outline how the behavioural science approach, together with use of qualitative methods, can contribute to AMS/AMR-related research in primary care, and thus enable more people to understand and use these approaches. First, we introduce the behavioural science approach and explain its relevance to qualitative AMS research. Second, we provide an overview of behaviour change ‘tools’ (theories, techniques). Next, we discuss how the behavioural science methods can be used. We illustrate it with examples of qualitative AMS studies in primary care, while also highlighting areas where this approach could be more utilized in the future. Finally, we summarize the main contributions of the behavioural approach to qualitative AMS research and discuss key implications and future directions for research and practice. The theories, methods and studies presented in this paper are not exhaustive; they have been selected to illustrate how behavioural science can be used and contribute to AMR/AMS research.

## Introducing behavioural science and qualitative methodologies in AMS research

### Overview of the behavioural science and behaviour change research

Behavioural science covers a range of disciplines and methodologies that focus on understanding human behaviour (i.e. an observable action), and then predicting, altering or promoting certain behaviours. With the growing burden of non-communicable diseases and evidence linking human behaviours to health, theories and research were developed in health psychology to understand and leverage behaviour change to improve health-related outcomes. The idea was to study behaviour change in a systematic and replicable way, and report behaviour change interventions precisely and comprehensively (using consistent terminology), similarly to scientific experiments, thus developing ‘a cumulative science of behaviour change’.^15–17^ Over decades many different behaviour change theories and models were developed that outline mechanisms/processes of psychological and behaviour change.^18^ This was enhanced by approaches to classify the content (‘active ingredients’) of behaviour change interventions and link them with change mechanisms.^15,16,19–21^ This, in turn, enabled accumulation of evidence from trials of behaviour change interventions on consistent and effective content/techniques to change behaviours.^21–26^ In parallel, methods and principles for developing and evaluating complex behaviour change interventions were developed and systematized.^27–31^ These approaches have been used to develop evidence on effective behaviour change interventions and techniques to prevent unhealthy (e.g. smoking^32^) and promote healthy (e.g. healthful diet and physical activity^33,34^) lifestyle behaviours. As tapering of effects and relapses to old behaviours are common following an initial behaviour change, lifestyle interventions increasingly focus on behaviour maintenance to ensure more sustainable impact.^35^

### Relevance of the behavioural science approach to AMR/AMS

Behavioural science methods and tools, and behaviour change theories and strategies, were largely absent from the early interventions to address AMS/AMR and continue to be underutilized in AMS/AMR research.^11,36–39^ However, improvements in AMS/AMR depend on supporting the behaviour change of individuals, groups and organizations. These sets of behaviours (actions) relate to antibiotic prescribing and use, preventing and managing/treating infections, and implementing/using AMS interventions. The behavioural science approach can contribute to understanding and promoting change in these behaviours, and in developing and evaluating behavioural AMS/AMR-related interventions. Hence, to address this discrepancy between the absence of behavioural science and its potential, there have been calls to promote the use of behavioural science in AMS/AMR research to understand behavioural determinants and develop effective interventions in primary care^9^ and secondary care.^7,8,40–42^ For example, Lorencatto *et al*.^41^ outlined four areas where behavioural and social science can contribute to AMS/AMR research: (i) defining the problem in behavioural terms and understanding the current behaviour in a particular context; (ii) adopting a theory-driven, systematic approach to intervention design; (iii) investigating implementation and sustainability of interventions in practice; and (iv) maximizing learning through evidence synthesis and detailed intervention reporting. Donisi *et al*.^40^ proposed to incorporate a behavioural science approach into: (i) assessment of the specific context needs; (ii) intervention design; (iii) implementation, communication and education; and (iv) follow-up and audits. The behavioural science approach is well-positioned to contribute to AMS/AMR research as it offers: theories and frameworks to help us understand behaviour change processes and their determinants; techniques to change behaviours and evidence on what works in behaviour change; and principles and methods to systematically develop, refine, implement and evaluate behaviour change interventions. We discuss these aspects in the subsequent sections.

### Compatibility of the behavioural science approach and qualitative methodologies

The behavioural science approach fits well with, and often relies on, qualitative methodologies. First, qualitative methods allow an exploratory approach that can help to identify relevant behaviours and to understand influences on behaviours and how best to support behaviour change. Second, qualitative content coding can be used to identify behaviour change techniques in intervention reports and accumulate evidence on effective strategies to change behaviours. Third, qualitative methods are particularly well-suited and recommended for developing, implementing and evaluating behaviour change interventions. Thus, combining behavioural and qualitative methods can contribute to better understanding and improving AMS-related behaviours in primary care and the community. In this paper we draw on examples of studies that combined these approaches. More details on how qualitative methodologies can be used in, and contribute to, the AMS/AMR research can be found elsewhere (M. Wanat, M. Santillo, A. J. Borek, C. C. Butler, S. Tonkin-Crine, ‘The value, challenges and practical considerations when conducting qualitative research on antimicrobial stewardship in primary care’, under review).^14,43^

## Overview of behaviour change tools

### Theories and models of behaviour change

A good behaviour change theory provides ‘a parsimonious, coherent explanation of phenomena and general predictions that can be compared against observation’ (Michie *et al*.,^18^ p. 23). A model is often considered a schematic representation (e.g. of a theory or phenomenon) but a distinction between theories and models is often blurred and the terms are sometimes used synonymously.^44^ Theories and models are important because they offer testable, falsifiable predictions, provide a common language to describe the phenomena, and help formulate and address questions and accumulate knowledge systematically.^18^ Therefore, they can inform development and evaluation of behaviour change interventions in systematic ways, and expand evidence on what works, when and how.

Over 83 behaviour change theories/models have been identified to date,^18^ and 100 behaviour change maintenance theories.^35^ Some theories are specific to particular behaviours (e.g. smoking, communication), populations (e.g. adolescents) and types of change (e.g. individual, social, technological). Table 1 briefly summarizes examples of theories of individual health-related behaviour, which have been used in interventions to change AMS/AMR-related behaviours. While behaviour change theories mostly focus on individual, intrapersonal change, there are also theories that relate to group-level behaviours (e.g. group dynamics^45^), interpersonal behaviour change processes^46,47^ (e.g. social comparisons,^48^ social influence^49^), and social change (e.g. social change theory^50^ on social norms influencing community and individual change, social action theory^51^ for population-level behaviour change). It is important and prudent to draw on this wealth of behaviour change theories in AMS/AMR research.

**Table 1.** Selected examples of behaviour change theories/models and their use in AMS interventions

Example behaviour change theories	Summary of proposed psychological and behaviour change mechanisms	Examples of potential and actual use in AMS interventions in primary care and the community
Health belief model (by Rosenstock)^102^	Health-related behaviour (change) is influenced by the perceived susceptibility to the health risk, perceived seriousness of the health risk and belief that perceived benefits of taking action outweigh the barriers to taking action. Other modifying factors include cues to action (internal, external), demographic variables, sociopsychological variables, structural variables (e.g. knowledge about the disease) and self-efficacy (i.e. belief about capability to take action; added later to the model).	To understand and promote behaviours of: patients to prevent infections (e.g. hand-washing) clinicians to reduce antibiotic prescribing to lower patients’ risks of side effects and drug-resistant infections e-Bug resources to improve children’s behaviours related to hand, respiratory and food hygiene^103^
Social learning theory (by Miller and Dollard^104^ and Bandura)^105^	Learning is influenced by drives (motivation for action), cues (stimuli that determines whether, when and where action is taken), responses and rewards. Learning occurs when a response to certain drives and cues is performed and rewarded (internally/externally). It also occurs through imitation of others. Bandura’s theory proposed that learning occurs through observing, modelling, imitating, and reactions of others, and is also influenced by interacting environmental and cognitive factors.	To understand and influence why, how and when: patients learn to expect antibiotics clinicians learn to prescribe antibiotics (unnecessarily) STAR intervention to increase use of communication strategies and reduce antibiotic prescribing by primary care clinicians^12^
Theory of planned behaviour (by Ajzen)^106^	Behaviour (change) is influenced by attitudes (positive/negative beliefs about the behaviour), subjective norms (perceptions of others’ beliefs about and approval of the behaviour), perceived behavioural control (belief of whether one is able to perform the behaviour, and of barriers/facilitators to its performance); these factors influence behavioural intentions (motivation or willingness to perform the behaviour), which then influences the behaviour.	To understand and influence intentions of: patients to consult (or not) for similar acute infections clinicians to reduce unnecessary antibiotic prescriptions and provide self-care advice GRACE-INTRO intervention to increase use of communication strategies and point-of-care tests, and reduce antibiotic prescribing in primary care^13,95^
Self-efficacy theory^107^ and social cognitive theory^108^ (by Bandura)	Behaviour (change) is influenced by people’s beliefs about being capable of that change (i.e. self-efficacy), which are influenced by information from: performance accomplishment (e.g. personal experience of success/failure), vicarious experience (of the behaviour or its observation), verbal persuasion and emotional arousal (e.g. stress). Social cognitive theory proposes that the behaviour, the environment and the personal and cognitive factors (including perceived self-efficacy) all interact and determine each other.	To understand and influence: patients’ self-efficacy for self-care behaviours clinicians’ self-efficacy for adhering to prescribing guidelines; environmental factors supporting adherence with guidelines (e.g. embeddedness within IT system) REDUCE study intervention to promote use of prescribing guidelines in primary care^109,110^
COM-B system (by Michie *et al*.)^53,111^	Behaviour (change) is influenced by three interacting elements: capability (physical, psychological); opportunity (physical, social); and motivation (automatic, reflective). Behaviour occurs when the motivation to engage with it is greater than motivation for alternative behaviours.	To understand and influence COM-B elements for: patients to perform self-care behaviours clinicians to comply with prescribing guidelines Antibiotic Guardian Youth programme to improve infection prevention-related behaviours^112^

### Behavioural determinants

Behavioural determinants are the types of factors that influence (facilitate or impede) behaviour and behaviour change. Behavioural determinants identified in behaviour change theories have been synthesized, organized and defined in the theoretical domains framework (TDF).^52^ The TDF is a list of 14 domains (types) of health-related determinants of individual behaviours (Table 2), which comprise 83 more specific constructs. It is an important and helpful ‘tool’ in the behavioural science approach because it integrates, simplifies and makes more accessible the many different constructs included across behaviour change theories. It provides common vocabulary that can be used to describe behavioural determinants in more abstract, general, and thus comparable terms. The TDF, as a framework, does not explain how these concepts may influence or lead to behaviour change, and which are or are not relevant and important for the targeted behaviour change. However, as these constructs were derived from theories, they can be reverse-linked with change mechanisms. After identifying important behavioural determinants in a target population and for a target behaviour, we can select the most appropriate intervention functions and behaviour change techniques (BCTs) to address them. The behaviour change wheel guide^53^ provides matrices that link the TDF domains with theoretically congruent COM-B elements, intervention functions (Table 2) and BCTs.^54^ For example, the TDF domain ‘knowledge’ can be addressed by intervention function ‘education’, ‘skills’ by ‘training’, whereas ‘beliefs about consequences’ can be addressed by interventions involving ‘education’, ‘persuasion’ and/or ‘modelling’.

**Table 2.** Behavioural determinants and intervention functions

Domains of behavioural determinants in the TDF^52^	Intervention functions from the behaviour change wheel^53^
Knowledge Skills Social/professional role and identity Beliefs about capabilities Optimism Beliefs about consequences Reinforcement Intentions Goals Memory, attention and decision processes Environmental context and resources Social influences Emotion Behavioural regulation	Education (providing information) Persuasion (communicating to induce feelings to prompt the behaviour) Training (imparting or practising skills) Modelling (providing examples to model or aspire to) Incentivization (offering incentives or rewards for the behaviour) Enablement [increasing means (e.g. opportunities, support) or reducing barriers to the behaviour] Environmental restructuring (using physical or social cues for action, e.g. prompts, physical materials) Restriction (setting rules for the behaviour) Coercion (creating an expectation of punishment or cost)

### Behaviour change techniques (BCTs)

BCTs are also a key element in the behaviour change ‘toolkit’. A BCT is ‘an observable, replicable, and irreducible component of an intervention designed to alter or redirect causal processes that regulate behaviour; that is, a technique is proposed to be an “active ingredient” of an intervention.^55^ Taxonomies of BCTs were derived from earlier classifications and descriptions of interventions, mostly targeting lifestyle behaviours.^21,55^ The most comprehensive BCT Taxonomy v1 comprises 93 types of BCTs, organized into 16 categories.^55^ Other taxonomies were developed specific to different health behaviours/outcomes, such as smoking cessation,^26^ sexual health^22^ or weight loss.^56,57^ Although to our knowledge there is currently no taxonomy of BCTs specific to AMS/AMR-related behaviours, the BCT Taxonomy v1 is a useful source of generic and commonly used BCTs.

BCT taxonomies are helpful because they provide ‘menus’ of BCTs that could be selected and used to facilitate behaviour change processes. However, they do not provide guidance on which BCTs may be helpful or effective at changing a particular determinant or behaviour, so they should be used with consideration of change mechanisms, i.e. which BCTs are theoretically congruent with different TDF domains and/or intervention functions.^53,54^ BCT taxonomies also help specify the content of interventions during intervention design and reporting, thus improving clarity and transparency of the ‘active ingredients’ in behaviour change interventions. Finally, they can be used to code the content (descriptions) of existing interventions and compare them in systematic reviews and meta-analyses, thus helping develop evidence on which techniques increase intervention effectiveness.^23,24,26,58^

## Using behavioural science and qualitative methods in AMS research in primary care

In this section we describe how the behavioural science methods and behaviour change tools can be used together with qualitative methods to target AMS-related behaviour change in primary care. We illustrate it with examples from our and others’ studies, and highlight areas for future contributions.

### 1. Identifying target behaviours and behavioural influences

Before we can address a health-related problem, the behavioural science approach stipulates that we first conduct a ‘behavioural diagnosis’ to develop a good understanding of what the problem is and identify behaviour(s) contributing to it and influences on these behaviours.^41,53^

When identifying target behaviours, it helps to do so precisely—that is, by specifying the target behaviour and type of change, how to perform it correctly, who needs to perform it, and when or in what context. Many AMS behaviours are actually complex *sets* of multiple behaviours (actions, steps) performed by different people and in different contexts. For example, infection prevention is a vague category that could include many potential behaviours (e.g. handwashing, covering a cough, avoiding contact); whereas washing hands is a specific behaviour but involves several steps to do it correctly (e.g. taking soap, hand movements, drying), at the right time, in the right context, and by the right person. It also helps to consider and specify what kind of behaviour change we aim for, for example, performance/discontinuation, increase/decrease or a change in an aspect of a behaviour (e.g. prescribing a different type of antibiotic). Moreover, we need to decide whether our intervention will target one behaviour (e.g. an automatic reminder of prescribing guidelines to prompt a change in the *typ*e of antibiotic prescribed), or if it will target multiple behaviours with multifaceted components (e.g. a training programme to increase the use of specific communication strategies and patient leaflets). Finally, it is important to specify the target population (i.e. whose behaviour needs to change, e.g. patients, doctors, administrative staff), and when or in what context (e.g. during or outside a consultation).

Facilitating change in modifiable, observable and specific behaviours that contribute to the identified issue is the main aim of behavioural interventions; whereas facilitating change in psychological/behavioural determinants is *how* that change can be achieved. Psychological concepts, such as intentions or motivation, are behavioural determinants and may not always correspond with actual behaviour; this is commonly referred to as the ‘intention-behaviour gap’.^59^ For example, one can be aware of the importance of a behaviour (e.g. handwashing) and motivated to perform it, but may still not perform it due to different types of barriers (e.g. environmental: lack of soap/space; social: observing others not washing their hands in a similar context; psychological/cognitive: forgetting/lack of habit).

After defining target behaviours, it is important to identify relevant behavioural determinants and influences. Influences are sometimes referred to as ‘barriers and facilitators’ to mean the factors that impede/prevent or facilitate/enhance the behaviour (change). It can also help to be mindful of a difference between influences on the behaviour versus influences on the behaviour change. For example, knowledge of prescribing guidelines influences antibiotic prescribing but alone will not necessarily influence a change in prescribing behaviour, whereas knowledge of a discrepancy between one’s prescribing and guidelines might. Behavioural determinants/influences can be also expressed in general, abstract terms using the TDF.^52^ For example, a belief that most acute infections resolve without antibiotics can be expressed using a TDF domain ‘beliefs about health consequences’.

Qualitative research can help identify target behaviours and behavioural influences. If relevant empirical research already exists, a review of qualitative studies can help identify these. For example, reviews of qualitative studies have synthesized influences on antibiotic prescribing for acute respiratory infections in primary care,^60–63^ and on parental views related to antibiotic prescribing for children in primary care.^64^ If sufficient or relevant research is lacking, conducting a qualitative study may help. For example, interviews and focus groups allow identification of participants’ perceptions and experiences of specific behaviours and behavioural influences.^65–69^ Other approaches, such as ethnographic^43,70^ and interaction analyses,^71,72^ can help identify patterns of behaviours and influences, rather than depending only on perceptions or recollections. Moreover, consulting with stakeholders and patient and public involvement (PPI) groups (e.g. comprising public and patient representatives) can help in multiple ways: to identify and select key target behaviours that are important and feasible to change and their determinants, to inform reviews and/or to design ethical and acceptable qualitative studies to explore the behaviours and determinants.

**Figure 1.**
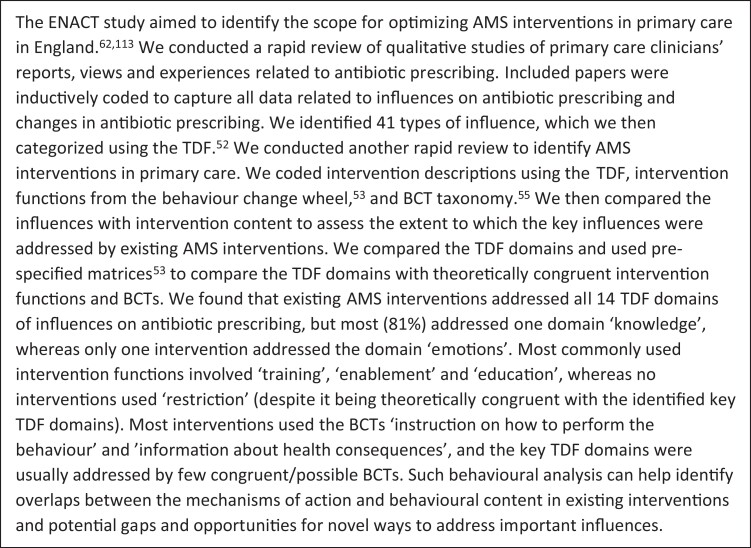
Example behavioural analysis of AMS-related influences and interventions in primary care.

Qualitative methods to identify behavioural influences can be informed and/or supported by the use the TDF^52^ or COM-B elements.^53^ For example, they can be used to inform semi-structured interview topic guides;^73,74^ this can be beneficial by ensuring that interviewers explore all the potential ways behaviours may be influenced. They can also be used to inform data analysis or interpretation in qualitative studies and syntheses of qualitative studies, including deductive^75^ and inductive (Figure 1)^62^ approaches; this can be beneficial by making specific types of influences more generalizable and comparable, and allowing linking them with behavioural mechanisms.

### 2. Developing and implementing AMS interventions

One of the main contributions of the behavioural science approach is in promoting and offering systematic, rigorous methods to develop behaviour change interventions. Many different approaches to intervention development exist; for example, a systematic methods overview and taxonomy identified eight *types* of approaches to developing health interventions (each with multiple specific methodologies).^29^ Some approaches are centred on behaviour change theory and evidence, e.g. the behaviour change wheel^53^ and intervention mapping;^76^ others focus on partnerships, co-production^77^ and target population (e.g. the person-based approach^78,79^). Behavioural theories and qualitative methods can contribute to most intervention development approaches, and can increase acceptability, feasibility and relevance of the interventions. For example, one of the barriers to clinicians changing their antibiotic prescribing is a perception that their actions have little role to play; theory-based interventions aimed to address these by providing reasons for change (the ‘why’) and feasible and time-efficient strategies (the ‘how’).^80^ The ‘Stemming the Tide of Antimicrobial Resistance’ (STAR) intervention was developed based on social learning theory and qualitative research as it aimed to address clinicians’ reasons for changing, increase their perceived importance of change, and their ability and perceived efficacy to change.^12,81^ The ‘Internet Training for Reducing antibiOtic use’ (GRACE-INTRO) intervention drew on the theory of planned behaviour as it aimed to influence clinicians’ attitudes, confidence and intentions to reduce antibiotic prescribing, and on qualitative research on influences and views of potential interventions.^60,82–84^

The person-based approach (PBA) is an example of an intervention development methodology that integrates the theory- and evidence-based approaches with qualitative methods with the target population and stakeholders.^78,79,85^ This helps ensure that interventions have clear intended mechanisms of change; are feasible to implement; and are acceptable, correctly understood and used by the target population. The PBA highlights the importance of identifying target behaviours and influences through (synthesizing or conducting empirical) qualitative research with the target population and stakeholder consultations. The influences are mapped to relevant theories and frameworks (e.g. COM-B^53^ or TDF^52^) to identify the behavioural determinants and theoretically congruent intervention functions. Relevant BCTs are selected to facilitate specific psychological and behaviour changes.^55^ The PBA (and other approaches) involves developing a programme theory that outlines how exactly the intervention is intended to work: its objectives, components/features and delivery methods, and how they are linked together into a mechanism of action. The programme theory may draw on an established behaviour change theory (or theories) or be unique to the intervention and target behaviours and population. A logic model is usually developed to diagrammatically summarize the programme theory.

**Figure 2.**
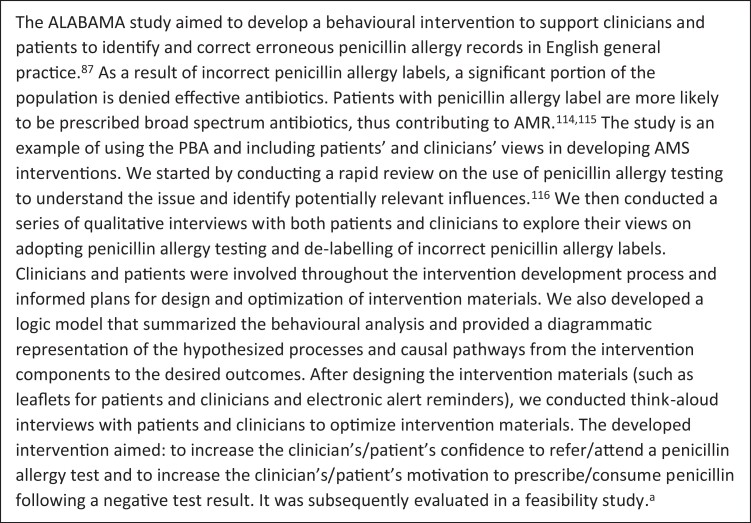
Example of developing an AMS intervention in primary care. ^a^M. Wanat, M. Santillo, U. Galal, M. Davoudianfar, E. Bongard, S. Savic, L. Savic, C. E. Porter, J. Fielding, C. Butler, S. H. Pavitt, J. A. T. Sandoe, S. Tonkin-Crine; ‘Mixed-methods evaluation of a behavioural intervention package to identify and amend incorrect penicillin allergy records in UK general practice’, under review.

The initial intervention (materials) can be then developed through (e.g. co-design) workshops and refined with ‘think-aloud’ interviews with the target users—these involve participants from the target population using the prototype/initial intervention while ‘thinking aloud’ and answering questions about design and content. They help identify ways to improve the intervention to make it more acceptable, easier to use and correctly understood, and thus more likely to be effective. During the different stages of intervention development research activities are accompanied by consultations with collaborators and stakeholders, and members of the PPI group. Using the PBA and qualitative methods is illustrated in developing AMS interventions in secondary^86^ and primary care (Figure 2).^87^

**Figure 3.**
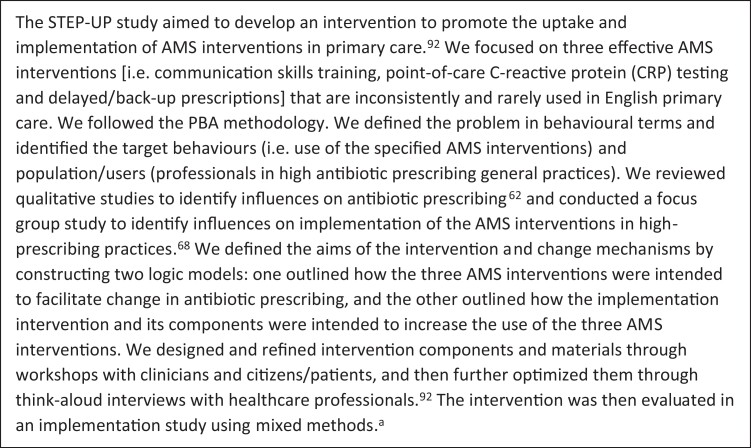
Example of developing an intervention to improve implementation of AMS interventions in primary care. ^a^S. Tonkin-Crine, M. Mcleod, A. J. Borek, A. Campbell, P. Anyanwu, C. Costelloe, M. Moore, A. Holmes, C. C. Butler, A. S. Walker and the STEP-UP team; ‘Supporting the use of three antibiotic stewardship strategies in high antibiotic prescribing general practices: an implementation study’, unpublished results.

A similar behavioural science approach can be also used to support and improve the implementation of existing AMS interventions in primary care. Implementation involves a complex set of behaviours; for example, it may require developing new practice protocols, introducing new practices, using new equipment or communicating a change in practice to the team. Implementation may require the change in individual, team or organizational behaviours and practices (e.g. ways of working together). Behaviour change tools can help facilitate change in implementation-related behaviours. For example, a guide outlines how the TDF can be applied to assess and address implementation issues.^88^ Moreover, there are theories and frameworks that are specific to outlining and facilitating implementation^89,90^and de-implementation processes.^91^ Interventions to address implementation issues can be developed using behavioural and qualitative methods and by drawing on behavioural and implementation theories/frameworks. For example, the PBA was used to develop an intervention to promote the implementation of evidence-based AMS interventions underused in English primary care (Figure 3).^92^

### 3. Evaluating behavioural AMS interventions

Evaluation and refinement of behaviour change interventions are important steps for achieving effective, feasible, acceptable and sustainable interventions and, in general, for accumulating evidence. The importance of evaluation and process evaluation, and of addressing a wide range of questions beyond only effectiveness, is highlighted in the Medical Research Council guidance on complex health interventions.^27,30,31^ These wider questions relate to the mechanisms of impact/action (i.e. how interventions facilitate change); delivery and implementation (i.e. what is delivered and how); and the wider context and system (i.e. what contextual/system factors influence the intervention delivery, implementation, mechanisms and outcomes). Qualitative methods are suitable for these different questions, can be integrated with social science and theory-based approaches,^93^ and, alongside quantitative research, allow triangulation of methods and findings.^94^

**Figure 4.**
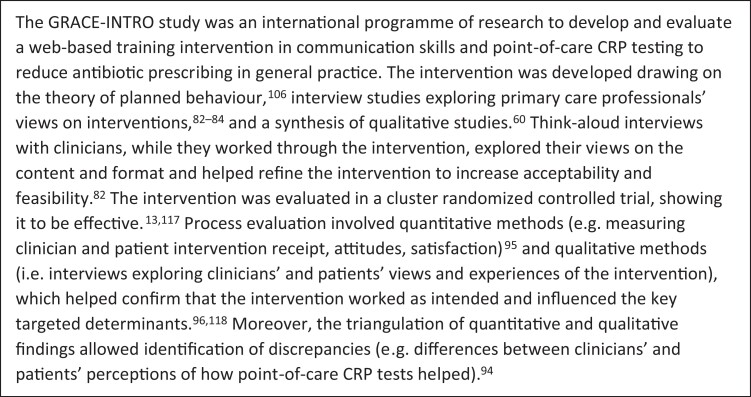
Example of using mixed methods to evaluate an AMS intervention in primary care.

Behavioural and qualitative approaches to process evaluations allow exploring and understanding the mechanisms of impact, whether the intervention worked in the intended way (i.e. compared with the programme theory), why it did not work or what unintended consequences it led to. The programme theory and logic model provide concepts and processes that should be explored in process evaluation. These usually include psychological change (e.g. in motivation, self-efficacy), cognitive change (e.g. in knowledge) and/or change in capabilities/skills related to performing the target behaviour. We can use qualitative methods to explore participants’ perceptions and experiences related to different types of change, whether and how interventions worked (or not), and which BCTs were seen as helpful (or not). For example, the mixed-methods process evaluation of the GRACE-INTRO intervention helped explore and confirm the change mechanisms (Figure 4).^95,96^

Behavioural and qualitative approaches can also contribute to exploring issues related to delivery, implementation and receipt of interventions, which may help explain outcomes and/or identify future refinements. Fidelity of delivery is the extent to which the core components of interventions are delivered as intended; good fidelity is critical for study validity.^97,98^ There are many aspects of fidelity, including intervention components, interventionist behaviours, participant comprehension and adherence.^98^ As delivery may involve intentional or unintentional changes to the intervention components delivered, fidelity of delivery should be assessed against the intervention theory/logic model that specifies the core intervention components and delivery methods. This is particularly important for interventions delivered by people (e.g. trainers, providers) who may need to be trained and provided with delivery manuals/protocols. Other important aspects include reach (i.e. the proportion of the target population who receive or are affected by the intervention),^99^ and receipt (i.e. the extent to which participants received the intervention, their engagement, comprehension and/or use of the intervention).^98^ These components are particularly important for digital or self-delivered interventions. Different methods can be used to ensure and assess intervention delivery and receipt.^97^ Qualitative methods may help to understand these issues through observations or analyses of recordings of the intervention being delivered, interviews or focus groups with participants and those delivering the intervention, or delivery checklists (with open questions about delivery issues or adaptations). For example, an intervention comprising a commitment poster for clinicians (pledging a commitment to prudent antibiotic prescribing) showed no significant effect on prescribing behaviour.^100^ Interviews with clinicians, as part of the process evaluation, showed suboptimal implementation and receipt of the intervention, which might partly explain the lack of effect; for example, some clinicians did not realize that they were making a pledge (e.g. their signatures were added to posters without clinicians reading them), and some thought the posters were supposed to influence patients’ not clinicians’ behaviours (S. Tonkin-Crine, A. Schneider, N. Herd, S. Michie, C. C. Butler, T. Chadborn, A. Sallis, ‘Implementing practice-level nudge interventions to encourage prudent antibiotic prescribing in general practice: a mixed-methods process evaluation’, under review).

The behavioural science approach and guidance for complex interventions also recognize the importance of exploring the role and influence of context in which the intervention takes place, and how it might affect the mechanisms, delivery/implementation and outcomes. Context may involve place, setting, team, organization or community, as well as the wider social, cultural, economic, regulatory, policy or political influences. For example, the behaviour change wheel model outlines seven categories constituting a wider policy and regulatory context (i.e. regulation, legislation, fiscal measures, guidelines, environmental/social planning and communication/marketing).^53^ Qualitative research can help us understand contextual factors. For example, interviews with professionals from general practices and clinical commissioning groups that explored the implementation and mechanisms of the Quality Premium (an intervention to improve antibiotic prescribing in English general practice) allowed us to identify contextual factors on the practice, local and national level that were perceived to influence antibiotic prescribing and engagement with AMS interventions.^69^ Another example involves interviews with professionals in general practices participating in the implementation study of the intervention described in Figure 3; the interviews helped to identify how the differences in the practice-level context (e.g. practice communication, leadership) influenced engagement with the intervention (S. Tonkin-Crine, M. Mcleod, A. J. Borek, A. Campbell, P. Anyanwu, C. Costelloe, M. Moore, A. Holmes, C. C. Butler, A. S. Walker and the STEP-UP team; ‘Supporting the use of three antibiotic stewardship strategies in high antibiotic prescribing general practices: an implementation study’, unpublished results).

## Contributions and implications of using the behavioural science in AMS research

**Figure 5.**
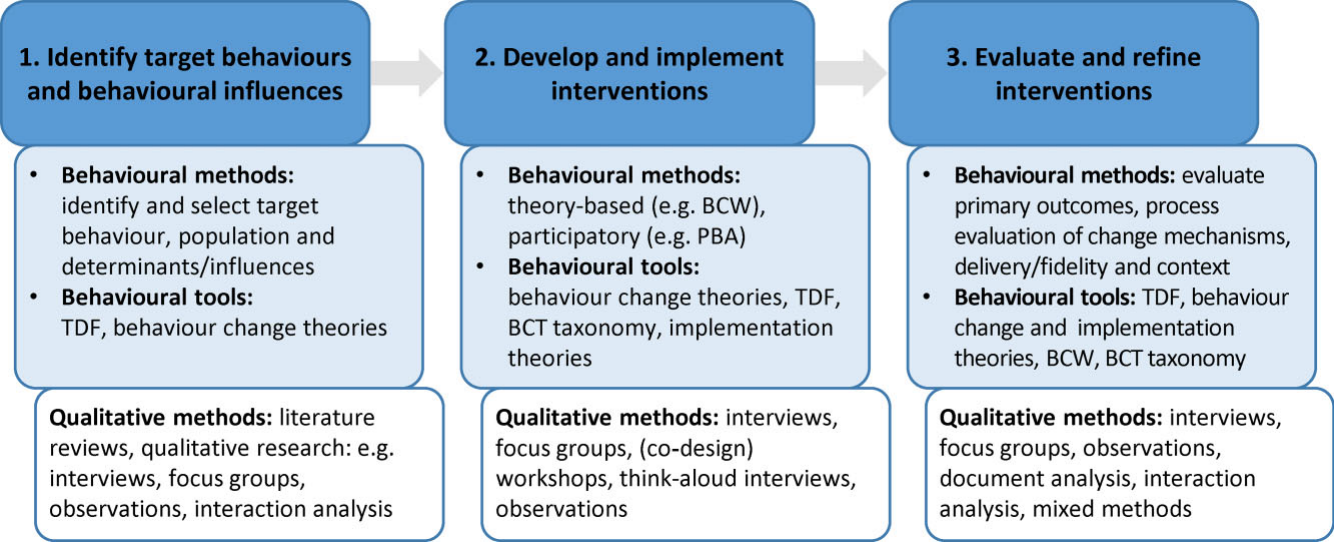
Contributions of the behavioural and qualitative methods to AMS/AMR research. BCW, behaviour change wheel; BCT, behaviour change techniques; PBA, person-based approach; TDF, theoretical domains framework.

Behavioural science and qualitative methods have contributed to AMS/AMR research and have the potential to improve existing and future AMS interventions: their capacity to affect relevant, important determinants; their acceptability, feasibility, reach and capacity to engage the target population; and their effectiveness and implementation outside research contexts. Figure 5 offers a summary of the described ways in which the behavioural science tools and methods, together with qualitative methods, can contribute to enhancing our knowledge of AMS/AMR issues and developing, improving, adapting and implementing AMS interventions.

The ambition for advancing health-related behaviour change research was to improve the scientific rigour, evidence and replicability of behaviour change interventions. Using behavioural theories and methodologies will help develop evidence of what works and how in AMS research. With the increasing threat of AMR consequences, it is critical to build on and improve existing knowledge and evidence.

Using the behavioural science approach with qualitative methods has implications for planning and conducting research and practice-based quality improvement projects. It means that those involved in such projects need to understand, or be trained in, the behavioural and qualitative methods. Projects can benefit from multidisciplinary teams including behavioural and qualitative researchers. Using the behavioural and qualitative methods has also methodological implications. It is important to consider carefully, and reflect on, how behavioural and qualitative methods are used together and what impact that might have on the process, data and findings. For example, consideration may be given to the potential impact of decisions about how theory is selected and used; who is involved in intervention development; which qualitative methodology is used (e.g. interviews allow identification of self-reported perceptions and experiences, whereas observations allow identification of actual behaviour); or whether deductive or inductive approaches to data analysis are used.

Behaviour change theories were mostly developed based on, and aimed at, health-related lifestyle behaviours. Some theories may be more or less relevant to AMS/AMR-related behaviours. Similarly, some BCTs may be not applicable to clinical and/or AMS/AMR behaviours, whereas other potential BCTs may have not been included in the existing taxonomies. Future research could develop or identify theories and BCTs specific to changing clinical and AMS/AMR-related behaviours. Moreover, it is important to develop theories, techniques and evidence on maintenance of promoted AMS-related behaviours (not only behaviour change), thus helping to improve sustainability of intervention effects.

The importance of transparent and comprehensive reporting of the content, mechanisms and active ingredients of behaviour change interventions seems now indisputable.^15–17^ Guidelines, such as TIDieR,^101^ promote better reporting of interventions (including e.g. theory, mechanisms, components, delivery, adaptations/modifications). Behavioural tools provide consistent, common vocabulary for behaviour change interventions. These tools and guidelines should be used when reporting AMS interventions too. Publishing reports of development and content of AMS interventions would also be helpful.

Finally, the behavioural science approach offers a perspective and a set of tools, out of many that might be relevant and helpful. Slowing down and mitigating AMR, a complex problem with different types of contributing/influencing factors, requires a multidisciplinary approach. Behavioural and social scientists can make important and helpful contributions as part of multidisciplinary teams, and may help develop relevant, effective/impactful, feasible, acceptable and implementable interventions.

In summary, our key recommendations for utilizing the behavioural science approach more fully in AMS/AMR research and practice include: ensuring a thorough understanding of behaviours and determinants/influences that contribute to the identified AMS/AMR-related problem *before* trying to change them; using more diverse qualitative methods to identify and understand behaviours and (behavioural and contextual) influences, especially through observations and interaction analyses; using and reporting behaviour change theories, frameworks and techniques more consistently in AMS interventions, and ensuring that each intervention has a programme theory and a logic model outlining the change mechanisms; involving target populations/users, stakeholders and PPI groups to guide/inform all stages of intervention development and evaluation; and including qualitative and mixed-methods process evaluations in studies piloting and evaluating behavioural AMS interventions. For behavioural scientists working on AMS/AMR, the next steps may need to involve developing behaviour change theories and techniques *specific* to AMS/AMR-related behaviours and contexts; further developing evidence on what works, how and in what contexts, when using different behaviour change strategies and types of interventions to address AMS/AMR-related problems; and promoting the implementation of effective behavioural AMS interventions.

## Conclusions

The behavioural science approach and qualitative methods fit well together and can contribute to AMS/AMR research. The behavioural science approach offers theories, frameworks and techniques related to changing human behaviours, and methods for developing behaviour change interventions. The qualitative approaches offer methodologies and methods for collecting, analysing and interpreting data from relevant target populations and stakeholders, and thus can help understand the behaviours and influences, develop acceptable, engaging and effective interventions, and evaluate them. These approaches were mostly absent from early AMS/AMR interventions,^11,36–38^ however their role in AMS has been increasingly recognized and promoted in secondary care.^7,8,40–42^ They now also have an important role to promote AMS and optimize antibiotic prescribing in primary care.^9^ We hope that this paper will enable more researchers and healthcare professionals to understand and use these approaches.

## Disclaimer

The views expressed in this publication are those of the authors and not necessarily those of the NIHR, UKHSA or the Department of Health and Social Care.

For the purpose of open access, the author has applied a Creative Commons Attribution (CC BY) licence to any Author Accepted Manuscript version arising.
